# Subthalamic Nucleus Deep Brain Stimulation Does Not Modify the Functional Deficits or Axonopathy Induced by Nigrostriatal α-Synuclein Overexpression

**DOI:** 10.1038/s41598-017-16690-x

**Published:** 2017-11-27

**Authors:** D. Luke Fischer, Fredric P. Manfredsson, Christopher J. Kemp, Allyson Cole-Strauss, Jack W. Lipton, Megan F. Duffy, Nicole K. Polinski, Kathy Steece-Collier, Timothy J. Collier, Sara E. Gombash, Daniel J. Buhlinger, Caryl E. Sortwell

**Affiliations:** 1 Department of Translational Science & Molecular Medicine, Michigan State University, Grand Rapids, MI USA; 2 MD/PhD Program, Michigan State University, Grand Rapids, MI USA; 3 Neuroscience Graduate Training Program, Michigan State University, Grand Rapids, MI USA; 4 Mercy Health Saint Mary’s, Grand Rapids, MI USA

## Abstract

Subthalamic nucleus deep brain stimulation (STN DBS) protects dopaminergic neurons of the substantia nigra pars compacta (SNpc) against 6-OHDA and MPTP. We evaluated STN DBS in a parkinsonian model that displays α-synuclein pathology using unilateral, intranigral injections of recombinant adeno-associated virus pseudotype 2/5 to overexpress wildtype human α-synuclein (rAAV2/5 α-syn). A low titer of rAAV2/5 α-syn results in progressive forelimb asymmetry, loss of striatal dopaminergic terminal density and modest loss of SNpc dopamine neurons after eight weeks, corresponding to robust human-*Snca* expression and no effect on rat-*Snca*, *Th*, *Bdnf* or *Trk2*. α-syn overexpression increased phosphorylation of ribosomal protein S6 (p-rpS6) in SNpc neurons, a readout of trkB activation. Rats received intranigral injections of rAAV2/5 α-syn and three weeks later received four weeks of STN DBS or electrode implantation that remained inactive. STN DBS did not protect against α-syn-mediated deficits in forelimb akinesia, striatal denervation or loss of SNpc neuron, nor did STN DBS elevate p-rpS6 levels further. ON stimulation, forelimb asymmetry was exacerbated, indicating α-syn overexpression-mediated neurotransmission deficits. These results demonstrate that STN DBS does not protect the nigrostriatal system against α-syn overexpression-mediated toxicity. Whether STN DBS can be protective in other models of synucleinopathy is unknown.

## Introduction

The gold-standard neurosurgical therapy for Parkinson’s disease (PD) is deep brain stimulation of the subthalamic nucleus (STN DBS). Current clinical practice is to consider DBS after adequate control of symptoms can no longer be achieved through pharmacotherapy. Since available pharmacotherapies are quite effective in early to mid-stage PD, the average patient undergoing DBS surgery is 12–14 years post diagnosis^1^.

The concept that STN DBS could potentially modify the course of PD has been explored in a handful of clinical studies. Although these clinical studies shared a post-diagnosis interval of ten years, results from these trials have yielded conflicting conclusions about disease-modification^2–6^. Of importance, the window of opportunity for disease modification in PD is within four years after diagnosis since striatal terminal loss is near complete by three to five years after diagnosis^7,8^. Only one study has enrolled the appropriate cohort; however, it was designed and statistically powered to evaluate the safety of STN DBS in early-stage PD and whether subjects would enroll^9^. As a result, no clinical trial has examined the disease-modifying potential of STN DBS at the time when nigrostriatal fibers are still present and can be protected.

Preclinical studies in parkinsonian animal models have examined the impact of STN DBS on nigrostriatal degeneration induced by either 6-hydroxydopamine (6-OHDA) or 1-methyl-4-phenyl-1,2,3,6-tetrahydropyridine (MPTP) in both rats and non-human primates^10–14^. STN DBS has been applied in these models prior to nigrostriatal degeneration as well as after nigrostriatal degeneration onset. STN DBS applied under these parameters results in varying degrees of neuroprotection of the dopamine neurons of the substantia nigra pars compacta (SNpc)^10–14^. These results demonstrate that STN DBS can provide significant neuroprotection from the degeneration resulting from acute oxidative stress elicited by neurotoxicant injection^15–23^. One major limitation of these studies is that the predictive validity of the PD neurotoxicant models is low. Indeed, numerous prospective therapeutics that protected nigral neurons against 6-hydroxydopamine and MPTP in preclinical studies have failed to translate to neuroprotection and symptomatic benefit in the clinic^24–26^. Therefore, evaluation of the neuroprotective potential of STN DBS in non-toxicant-based PD animal models is warranted to allow for more definitive validation.

Another major limitation of previous STN DBS studies examining neuroprotection against neurotoxicants is that none have observed sparing or reinnervation of striatal dopaminergic terminals^10–14^. In order for STN DBS-mediated neuroprotection to provide meaningful symptomatic benefits, the maintenance or restoration of dopaminergic innervation to the striatum must occur. Indeed, detailed post-mortem analysis confirms that loss of striatal dopaminergic innervation is a critical early event in PD and precedes and exceeds degeneration of SNpc cell bodies^8^. Therefore, the neuroprotective potential of STN DBS is optimally evaluated at both the level of the SNpc and the striatum.

**Figure 1:**
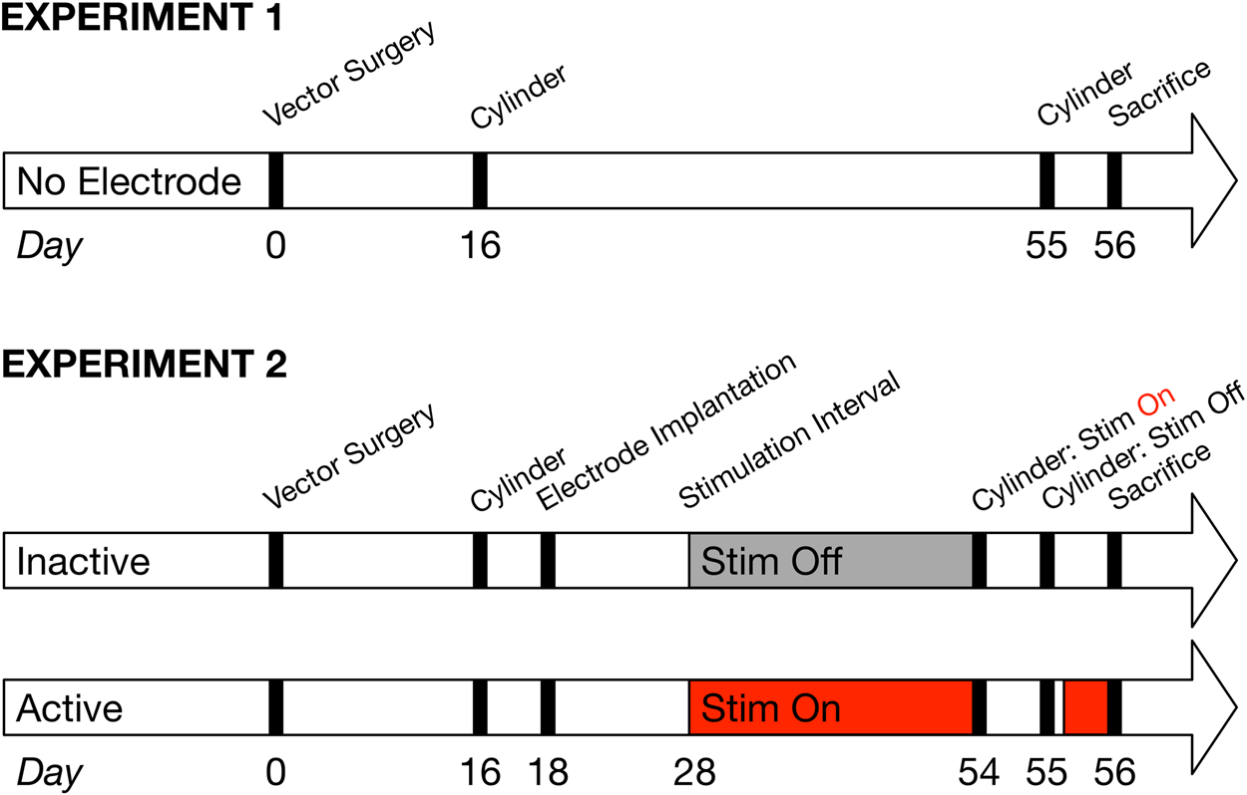
Experimental Design. Rats in Experiment 1 (n = 5) underwent intranigral administration of viral vector and were assessed using the cylinder task at 16 and 55 days after surgery. Rats in Experiment 2 (n = 12) underwent the same viral vector surgery and cylinder testing at Day 16 as in Experiment 1, but on Day 18 were implanted with an electrode in the STN. Rats were randomly assigned to receive continuous stimulation through Day 54 (‘Active’, n = 6) or to not have the electrode activated (‘Inactive’, n = 6). On Day 54, the cylinder task was conducted, and afterward, stimulation was ceased for 24 hours for forelimb asymmetry examination in the stimulation ‘off’ state at Day 55. After the cylinder task was completed on Day 55, stimulation was restarted for Active rats and continued until sacrifice. All rats in Experiments 1 and 2 were sacrificed on Day 56.

Models incorporating the overexpression of alpha-synuclein (α-syn) comprise another category of rodent models that display parkinsonian behavioral deficits, nigrostriatal terminal loss and degeneration of SNpc neurons. A large body of evidence points to the involvement of α-syn in PD, including the fact that point mutations and multiplications of the *Snca* gene, which encodes the α-syn protein, have been linked to the onset of familial forms of PD^27–29^. The subsequent discovery of α-syn in the hallmark protein aggregations known as Lewy bodies and dystrophic neurites of PD links α-syn to sporadic forms of the disease^30^. One preclinical PD model that has been extensively explored in our laboratory, and in others, is viral vector-mediated nigrostriatal overexpression of wildtype human α-syn^31–33^. This approach drives elevated α-syn expression levels within the nigrostriatal system specifically, resulting in α-syn aggregation, dopaminergic terminal dysfunction and loss, motor impairments and degeneration of SNpc neurons^33^. The magnitude and time course of these effects is dependent on α-syn expression levels^32^. In the present experiment, we explored the neuroprotective potential of STN DBS against the nigrostriatal toxicity that results from modest α-syn overexpression (experimental design in Fig. 1). Our choice of using modest α-syn overexpression rather than more marked α-syn overexpression was intended to more closely approximate α-syn levels in sporadic PD. We recently demonstrated that modest overexpression of α-syn more closely resembles the transcriptional signature observed in SNpc neurons in sporadic PD, whereas higher levels of overexpression produce downregulation of multiple trophic signaling models, downregulation that is not observed in sporadic PD^34,35^. Therefore, we contend that STN DBS-mediated neuroprotection in the context of modest α-syn overexpression will provide more predictive validity for potential neuroprotection in sporadic PD.

## Results

### Experiment 1: Effects of Nigrostriatal α-syn Overexpression

#### Recombinant adeno-associated virus pseudotype 2/5 (rAAV2/5) efficiently drives human wildtype α-syn expression in the nigrostriatal system

**Figure 2:**
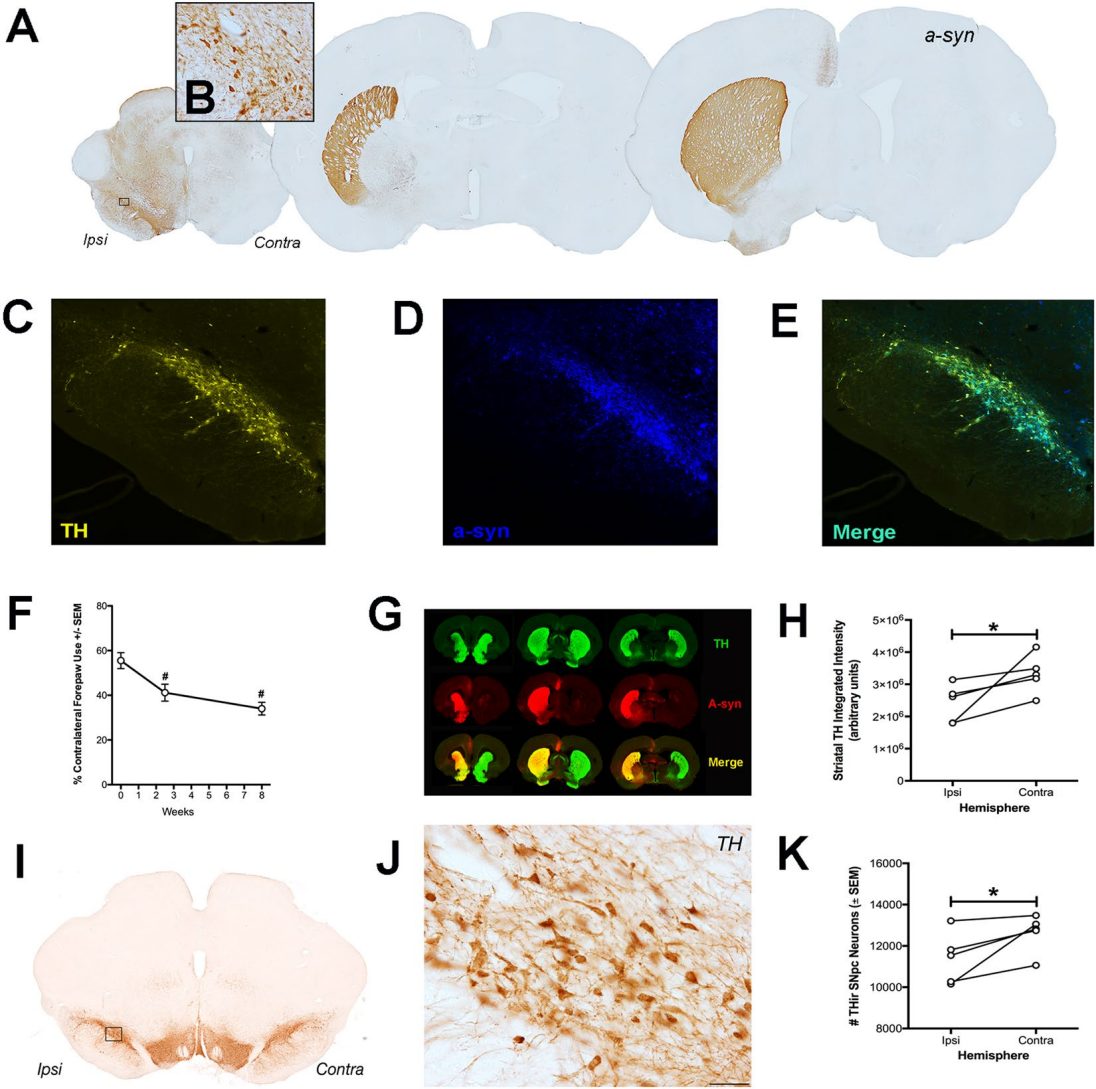
Overexpression of α-Syn Vector Model Recapitulates Early-Stage PD. Intranigral administration of rAAV2/5-α-syn (n = 5) results in unilateral transgene expression in the nigrostriatal system when examined for hu-α-syn immunoreactivity (**A**,**B**,**D**,**G**) and TH immunoreactivity (**C**,**G**) with extensive co-localization (**E**,**G**). Overexpression of α-syn results in modest but significant forepaw use asymmetry at sixteen days and eight weeks (**F**, *p* = 0.006 and *p* = 0.005, respectively), as well as robust, unilateral striatal α-syn immunoreactivity (*p* < 0.001) and a ~25% reduction in striatal TH immunoreactivity (**G** and **H**, *p* = 0.042). Overexpression of α-syn over eight weeks resulted in a significant 10% decrease of THir SNpc neurons in this specific cohort (**I**–**K**, *p* = 0.0258). Scale bar in **J** is 50 μm.

Rats in *Experiment 1* received intranigral injections of rAAV2/5 α-syn without electrode implantation (No Electrode treatment group) to allow for the initial characterization of the resulting synucleinopathy (Fig. 1). Our previous work identified a range of rAAV2/5 α-syn titers that result in varying levels of α-syn overexpression and corresponding differences in the magnitude of nigrostriatal tyrosine hydroxylase (TH) neuron degeneration at 8–12 weeks post injection^32^. In the present experiment, we modified these rAAV2/5 α-syn parameters with the intent of moderately overexpressing α-syn. α-Syn overexpression in the nigrostriatal system was verified using immunohistochemistry spanning the nigrostriatal axis (Fig. 2A,B), dual-label immunofluorescence in the SNpc (Fig. 2C–E) and near infrared immunofluorescence in the striatum (Fig. 2G).

#### Nigrostriatal α-syn overexpression results in significant contralateral forelimb akinesia

Contralateral forelimb akinesia was measured at baseline, prior to α-syn overexpression, sixteen days after vector surgery and at the end of eight weeks. Nigrostriatal α-syn overexpression resulted in significant contralateral forelimb deficits over time (F_(2,8)_ = 16.607, *p* = 0.001). Rats injected with rAAV 2/5 α-syn displayed less contralateral forelimb use at sixteen days (*p* = 0.006) and at eight weeks (*p* = 0.005) compared to baseline, indicating a functional deficit that emerges over time (Fig. 2F). Although contralateral forelimb deficits were slightly more pronounced at eight weeks (≈20% reduction from baseline) compared to sixteen days (≈15% reduction from baseline), no significant differences were observed between the two time points (*p* > 0.05).

#### Nigrostriatal α-syn overexpression results in decreased striatal TH immunoreactivity but does not impact dopamine tissue content in the striatum

The density of THir terminals and degree of human α-syn immunoreactivity was quantified in the striatum (Fig. 2G,H). As expected, human α-syn immunoreactivity was markedly elevated in the striatum ipsilateral to rAAV 2/5 α-syn injection compared to the contralateral striatum (t_(4.062)_ = 10.768, *p* < 0.001). Within the ipsilateral striatum significantly less TH immunoreactivity (≈25% reduction) was observed eight weeks after rAAV 2/5 α-syn injection as compared to the contralateral striatum (Fig. 2H; t_(4)_ = 2.479, *p* = 0.0341). However, this level of denervation was not adequate to alter total striatal dopamine or dopamine metabolite content that remained unaffected by unilateral α-syn overexpression (*p* > 0.05, Table 1).

**Table 1:** Levels of Striatal Monoamines and Metabolites.

	Ipsilateral	Contralateral
DA	144.9 ± 17.2	124.0 ± 10.3
HVA	10.7 ± 1.5	8.9 ± 0.8
DOPAC	13.3 ± 1.6	11.2 ± 1.0
HVA/DA	0.073 ± 0.003	0.072 ± 0.002
5-HT	12.9 ± 0.8	12.3 ± 0.3
5-HIAA	8.7 ± 0.7	7.7 ± 0.7

Levels of DA, HVA, DOPAC, 5-HT, 5-HIAA and the HVA to DA ratio were measured in the striatum of α-syn overexpressing rats (n = 5). No significant differences were observed in the levels of any of the monoamines examined between hemispheres (*p* > 0.05). Values are mean ± SEM in ng**/**mg protein except in the case of HVA/DA since it is unitless.

#### Modest nigrostriatal α-syn overexpression produces a small, significant decrease in THir SNpc neurons

Unbiased stereological quantification was used to assess survival of TH immunoreactive (THir) neurons within the SNpc ipsilateral and contralateral to rAAV 2/5 α-syn injection. The contralateral SNpc possessed 12,626 ± 411 THir neurons compared to 11,401 ± 561 remaining THir neurons in the ipsilateral, rAAV 2/5 α-syn injected hemisphere (≈10% reduction; Fig. 2I–K), a decrease that reached statistical significance (t_(4)_ = 2.747, *p* = 0.0258). These data suggest that the magnitude of nigral THir neuron loss induced by eight weeks of this level of α-syn overexpression is minimal.

#### Nigrostriatal α-syn overexpression does not alter expression of trkB or BDNF mRNA in the SNpc

**Figure 3:**
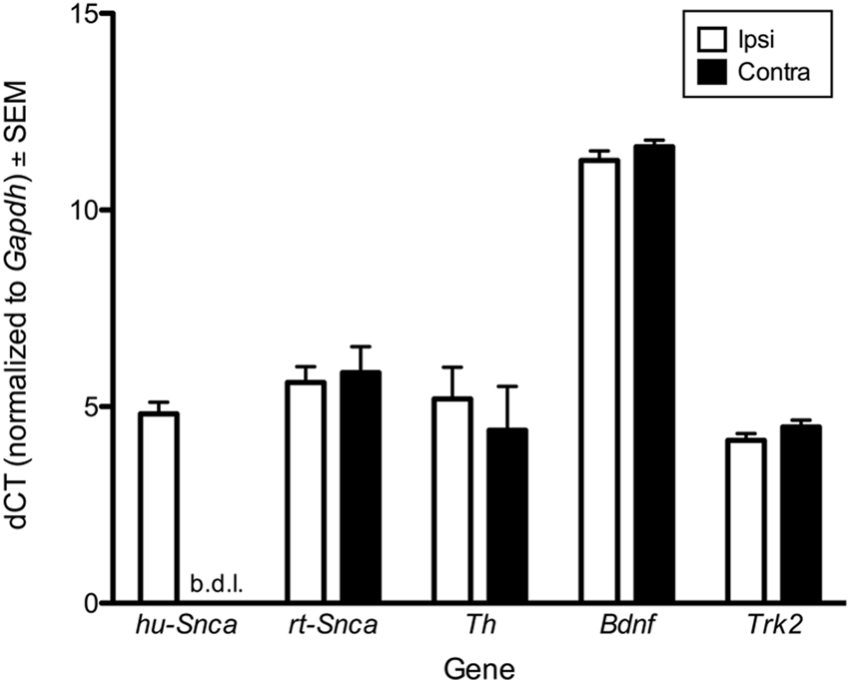
Overexpression of α-syn Does Not Alter SN Gene Expression. qPCR of the SN (ipsilateral vs. contralateral hemispheres) in the No Electrode group (n = 5) was conducted for the following transcripts: human *Snca* (hu-*Snca*, to confirm transduction), rat *Snca* (rt-*Snca*), *Th*, *Bdnf* and *Trk2*. No significant differences between hemispheres were revealed for rt-*Snca*, *Th*, *Bdnf* or *Trk2* (*p* > 0.05). b.d.l. = below detectable limits.

In previous studies, we have observed that STN DBS is associated with a significant increase in brain-derived neurotrophic factor (BDNF) in the nigrostriatal system^36^ and that BDNF-trkB signaling is required for STN DBS-mediated neuroprotection from 6-OHDA^37^. Further, it has previously been reported that marked nigrostriatal α-syn overexpression results in decreased mRNA expression of genes associated with neurotrophic signaling, including trkB and BDNF^35^. Therefore, to investigate whether the level of nigrostriatal α-syn overexpression associated with the present transduction parameters similarly altered gene expression of BDNF signaling molecules, we analyzed the SNpc expression (ipsilateral vs. contralateral hemispheres) of the following transcripts: human *Snca* (hu-*Snca*, to confirm transduction), rat *Snca* (rt-*Snca*), *Th*, *Bdnf* and *Trk2*, the gene for trkB (Fig. 3). As expected, rAAV 2/5 α-syn injection resulted in robust hu-*Snca* expression in the SNpc ipsilateral to injection using qPCR. However, no significant differences between hemispheres were observed for rt-*Snca*, *Th*, *Bdnf* or *Trk2*. These data suggest that the modest human α-syn transduction parameters used in the present experiment do not modify BDNF-trkB signaling at the transcript level, in contrast to the effects of more marked human α-syn overexpression^35^.

#### Nigrostriatal α-Syn overexpression increases phosphorylated ribosomal protein S6 (p-rpS6)

**Figure 4:**
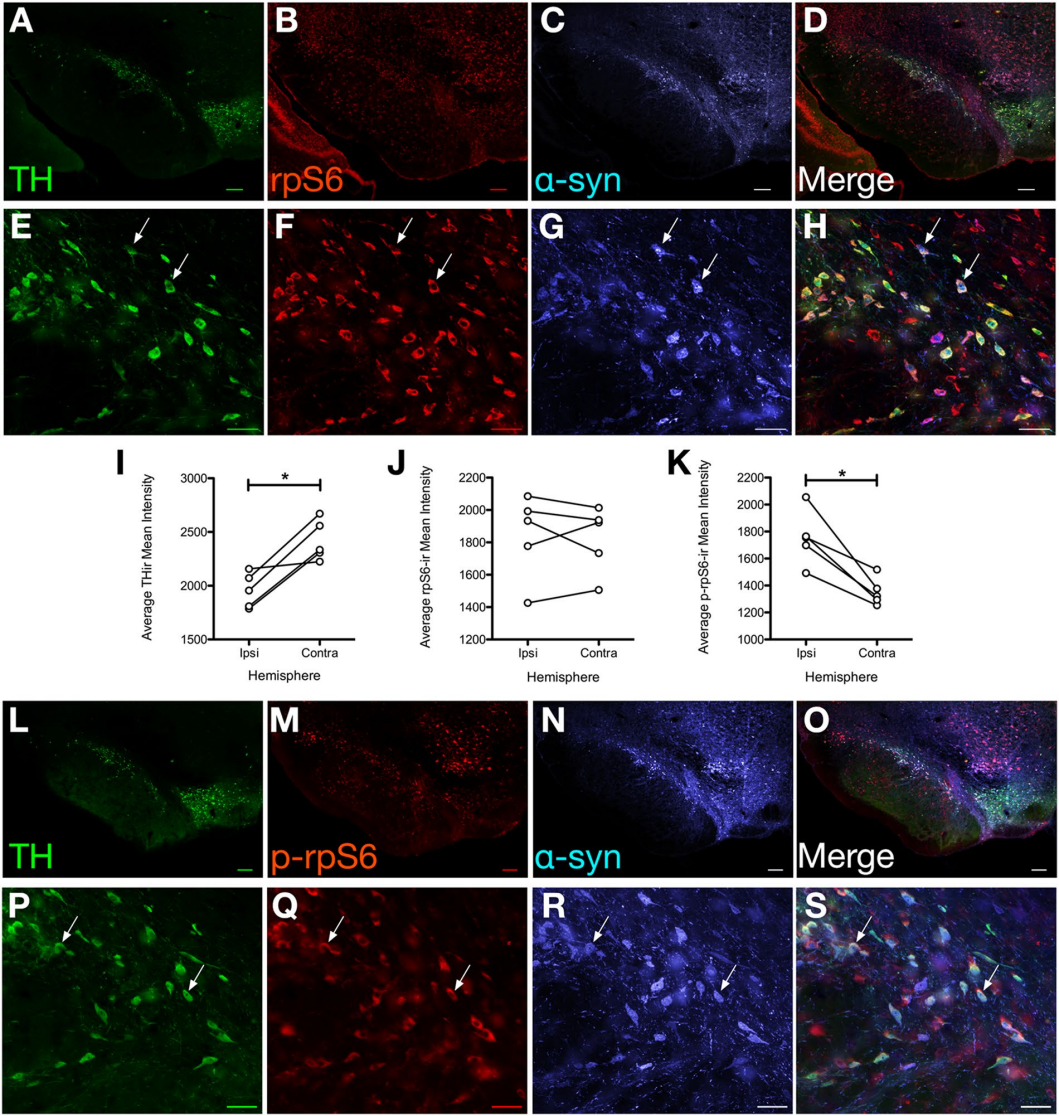
Effects of α-syn Overexpression on Total and Phosphorylated Levels of rpS6. Representative, low- and high-magnification images (**A**–**D** and **E**–**H**, respectively) from the SNpc ipsilateral to rAAV2/5 α-syn injection displaying triple label immunofluorescence for tyrosine hydroxylase (TH, green, **A**,**E**), ribosomal protein S6 (rpS6, red, **B**,**F**) and human alpha-synuclein (α-syn, blue, **C**,**G**). Merged images showing numerous SNpc neurons co-expressing all three proteins are shown at low (**D**) and high (**H**) magnification. From the same rat in an adjacent section, the ipsilateral SN is shown at low and high magnification for TH (**L**,**P**), p-rpS6 (**M**,**Q**), human α-syn (**N**,**R**) and merged images (**O**,**S**). Quantification of the No Electrode group (n = 5) was assessed between hemispheres to determine the effect of α-syn overexpression on TH (**I**), rpS6 (**J**) and p-rpS6 (**K**). A difference between hemispheres was observed for TH (**I**, *p* = 0.003) and p-rpS6 (**K**, *p* = 0.004). Scale bars are 200 μm and 50 μm for low- and high-magnification images, respectively.

Phosphorylation of ribosomal protein S6 (rpS6) can serve as a downstream marker of trkB activation following STN DBS and is associated with neuroprotection^37^. However, previous work has demonstrated that marked α-syn overexpression can decrease phosphorylation of rpS6 as well as phosphorylation of other mediators of trophic factor signaling^35^. In order to examine the impact of modest α-syn overexpression on both total rpS6 (rpS6) and phosphorylated rpS6 (p-rpS6) triple-label immunofluorescence for TH, hu-α-syn and either rpS6 or p-rpS6 was quantified in individual α-syn transduced SNpc DA neurons (Fig. 4). THir SN neurons expressing hu-α-syn were significantly less immunoreactive for TH (t_(4)_ = 4.606, *p* = 0.01, Fig. 4I) and exhibited significantly more p-rpS6 immunoreactivity (t_(4)_ = 4.867, *p* = 0.0082; Fig. 4K). However, overexpression of α-syn had no significant effect on levels of rpS6 (*p* > 0.05; Fig. 4J).

#### Experiment 1 Summary

Collectively, these results demonstrate that the intranigral rAAV 2/5 α-syn transduction parameters used in the present experiment lead to significant behavioral deficits in contralateral forelimb use, significant loss of THir innervation within the ipsilateral striatum and a modest significant loss of THir neurons in the ipsilateral SNpc. Under these transduction parameters α-syn overexpression has no effect on BDNF-trkB transcript levels; however, a significant increase in phosphorylated rpS6 is observed within α-syn overexpressing THir neurons. These findings suggest that BDNF-associated signaling pathways remain intact at the transcript level and appear already activated by α-syn overexpression. The parameters of this model are useful for testing the impact of STN DBS on early-stage, α-syn-induced nigrostriatal pathology.

### Experiment 2: Effects of STN DBS in the Context of Nigrostriatal α-syn Overexpression

#### Neither STN electrode implantation nor active STN stimulation alter α-syn transduction levels in the rat AAV2/5 α-syn model

**Figure 5:**
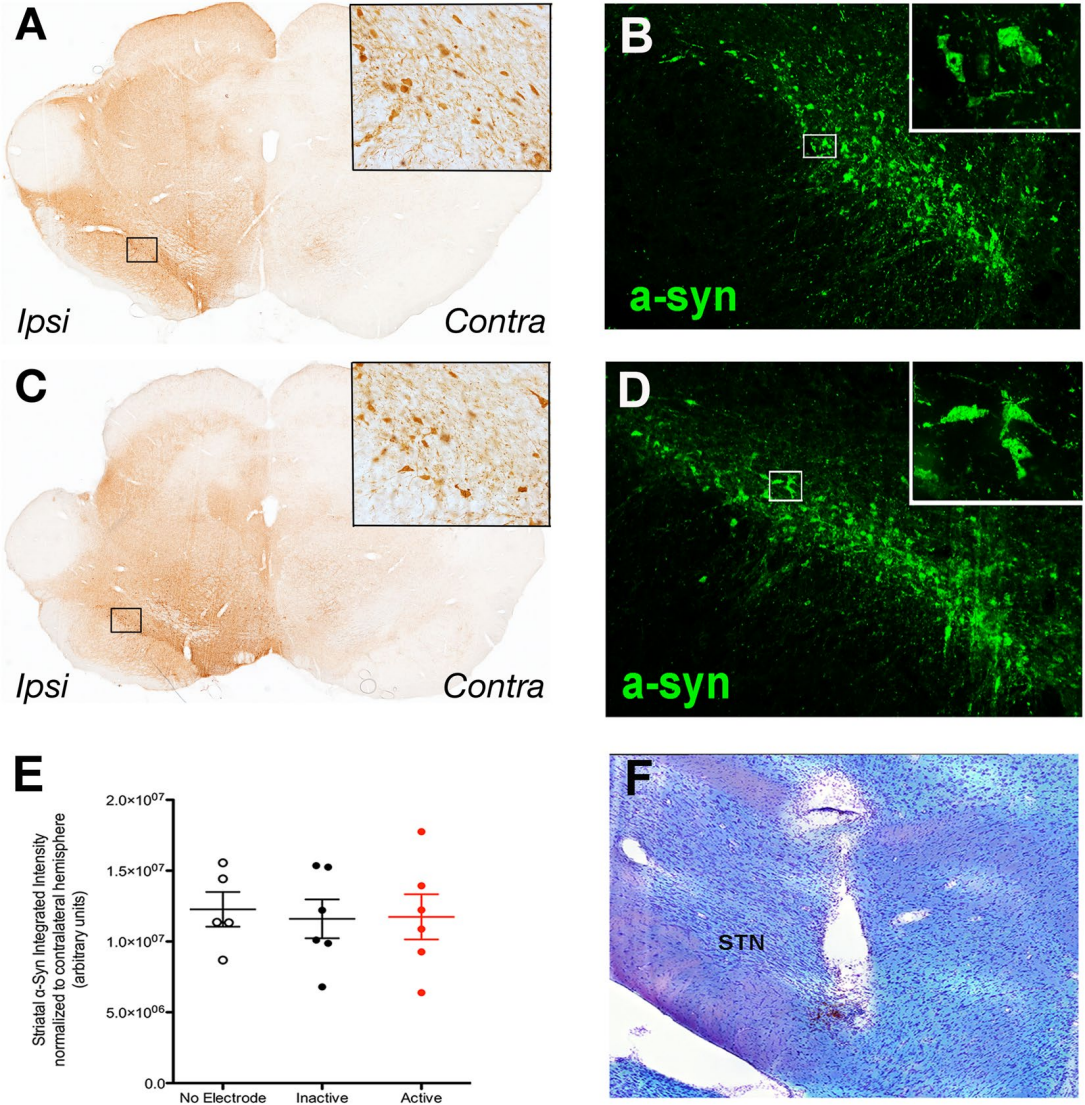
STN Stimulation Does Not Alter α-syn Transgene Expression. Active (**A**,**B**) and Inactive (**C**,**D**) groups showed robust α-syn transgene expression as revealed by immunohistochemical brightfield (**A**,**C** with high-magnification inset) or immunofluorescent **(B**,**D** with high-magnification inset) labeling methods in the SNpc. Striatal α-syn immunoreactivity in the ipsilateral hemisphere was not significantly different between the No Electrode, Inactive and Active groups (n = 5, 6 and 6, respectively; **E**, *p* > 0.05). Electrode-implanted rats included in this study were examined post mortem for electrode placement targeting the STN (**F**). The tract previously occupied by the electrode is appreciable dorsal and slightly medial to the STN.

Rats in *Experiment 2* received intranigral injections of the identical rAAV2/5 α-syn titer as described in *Experiment 1* and in addition, were surgically implanted with stimulating electrodes into the STN on Day 18 following vector injection (Fig. 1). Between Days 28–54, rats received either continuous ipsilateral STN stimulation (‘Active’ group) or no stimulation (‘Inactive’ group). Qualitative examination of α-syn immunoreactivity in the ipsilateral SNpc of the Inactive and Active groups revealed robust human WT α-syn expression (Fig. 5A and B) that appeared identical to the level of α-syn expression in the No Electrode group in *Experiment 1* (Fig. 2A,B and D). As in the No Electrode group, intranigral rAAV 2/5 α-syn injection resulted in a significant elevation in human α-syn immunoreactivity (F_(3,23)_ = 41.183, *p* < 0.001). Specifically, human α-syn immunoreactivity levels were significantly higher in the ipsilateral striatum of both the Active and Inactive groups, compared to their respective contralateral hemispheres (*p* < 0.001); however, there was no difference due to treatment within a hemisphere (*p* > 0.05). Further, when human α-syn immunoreactivity levels in Active or Inactive ipsilateral striatum were compared, there was no significant effect of either electrode implantation or Active STN stimulation on levels of human α-syn immunoreactivity (*p* > 0.05, Fig. 5C). STN electrode placement was verified, as described previously^10^ (Fig. 5D), and a small number of rats with misplaced electrodes were not included in the analysis. These results show that neither the implantation of electrodes nor stimulation of the STN interfere with the level of human α-syn overexpression, a critical quality control step in order to allow for the effects of STN DBS on α-syn-mediated toxicity to be evaluated.

#### Long term STN DBS does not result in functional neuroprotection ‘OFF’ stimulation

**Figure 6:**
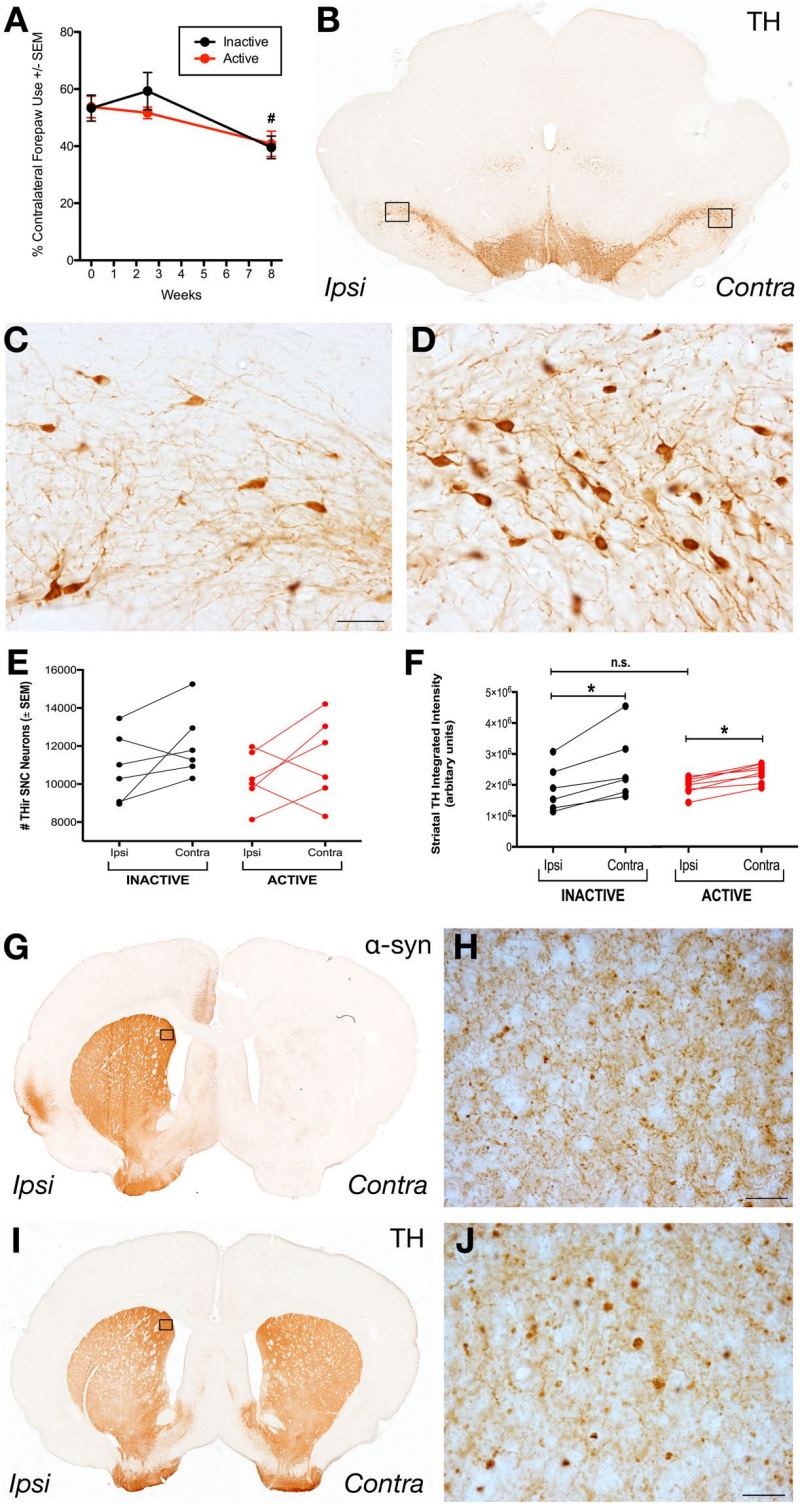
STN Stimulation Does Not Alter Symptom Progression Nor Neuropathology. Both Inactive and Active groups (n = 6 per group) exhibited forepaw use asymmetry after eight weeks compared to baseline or just prior to electrode placement surgery (**A**, *p* = 0.004 and *p* = 0.011, respectively); though, there was no interaction between group and time, so stimulation did not significantly alter the development of symptoms at eight weeks. THir SNpc neurons were not different between Active (shown in **B** at low magnification and in **C** and **D** at high magnification for the ipsilateral and contralateral SNpc, respectively) and Inactive groups (**E**, *p* = 0.314). (**F**) Striatal THir was not significantly different between groups (*p* > 0.05) but was significantly different between hemispheres for Inactive (*p* = 0.0002) and Active groups (*p* = 0.0087). Inspection of the α-syn-ir or THir striatum (**G**,**H** and **I**,**J**, respectively, for low and high magnification photomicrographs of an Active rat) did not reveal any apparent differences to a blinded investigator in α-syn-ir aggregates (**G**,**H**) or THir axonal swellings (**I**,**J**) between Active and Inactive rats. Scale bars are 50 μm.

The ability of continuous STN DBS stimulation between Days 28–54 to alter contralateral forelimb deficits elicited by unilateral intranigral rAAV2/5 α-syn injection was assessed. Contralateral forelimb akinesia was quantified in rats that received continuous Active stimulation between Days 28–54 or Inactive, electrode-implanted rats that received no stimulation over the same interval. Forelimb use was assessed at baseline (before vector injection), before electrode implantation (Day 16) and on Day 55, with stimulation turned off for a period of 24 hours. Forelimb function was assessed in the stimulation ‘OFF’ state to determine whether the functional neuroprotection from prolonged stimulation was distinct from the reversal of forelimb deficits normally associated with acute STN stimulation in unilaterally lesioned rats^11,36,38–47^. Similar to effects of nigrostriatal α-syn overexpression observed in rats without electrodes in *Experiment 1* (Fig. 2F), a statistically significant deficit in contralateral forepaw use over time was observed (F_(2,20)_ = 9.380, *p* = 0.001). After post hoc comparisons, contralateral forelimb use in the OFF state at eight weeks was impaired significantly when compared to baseline (*p* = 0.014) or at 16 days (*p* = 0.004). However, no significant interaction between time and stimulation treatment group was observed (*p* > 0.05, Inactive vs. Active, Fig. 6A). These OFF stimulation results indicate that neither STN electrode implantation nor continuous STN stimulation altered the progressive impairment of contralateral forelimb use resulting from nigrostriatal α-syn overexpression.

#### Continuous Active STN DBS does not protect against α-syn mediated loss of SNpc neurons

In *Experiment 1* we determined that eight weeks of nigrostriatal α-syn overexpression produced a small but significant decrease in SNpc THir neurons (Fig. 2K). Stereological analysis of SNpc THir neurons in Active and Inactive rats in *Experiment 2* revealed a similar trend in α-syn overexpression-induced nigral degeneration that did not reach statistical significance. Specifically, no significant differences between the number of THir SNpc neurons in the ipsilateral α-syn overexpressing SNpc versus the contralateral SNpc in either Inactive (t_(5)_ = 1.792, *p* > 0.05) or Active rats (t_(5)_ = 4.175, *p* > 0.05, Fig. 6E) were observed. Further, comparisons between THir SNpc neurons ipsilateral to α-syn overexpression in Active and Inactive rats revealed that STN DBS did not alter the number of THir SNpc neurons (F_(3,20)_ = 0.6956, *p* > 0.05, Fig. 6E). In order to examine the effect of electrode implantation on SNpc THir neurons all ipsilateral (to α-syn and to DBS lead), SNpc THir Active and Inactive counts were combined and compared to all contralateral SNpc THir Active and Inactive counts. With this enhanced statistical power, significantly fewer SNpc THir neurons were detected ipsilateral to rAAV 2/5 α-syn injection compared to the contralateral SNpc (t_(11)_ = 2.106, *p* = 0.0295), revealing an ≈8% decrease in the ipsilateral SNpc due to α-syn overexpression. These results demonstrate that electrode implantation may increase the variability of α-syn mediated nigral degeneration; however, comparison of ipsilateral THir SNpc neurons between Active and Inactive rats demonstrates that the modest α-syn-mediated loss of THir neurons in the SNpc is not affected by STN DBS.

#### Continuous Active stimulation does not protect against α-syn-mediated axonopathy

We next analyzed whether long-term STN stimulation protected against the 25% loss of striatal TH immunoreactivity induced by nigrostriatal α-syn overexpression in this model of early-stage parkinsonian pathology (Fig. 2H). Significant loss of striatal TH immunoreactivity was observed in the striatum ipsilateral to α-syn overexpression in both Inactive (t_(7)_ = 6.986, *p* = 0.0002) and Active rats (t_(5)_ = 4.175, *p* = 0.0087, Fig. 6F). However, STN DBS did not alter this pattern of α-syn overexpression-induced striatal denervation, as no significant differences in striatal TH immunoreactivity were observed in the ipsilateral striatum of Active rats compared to the ipsilateral striatum of Inactive rats (F_(3,24)_ = 1.821, *p* > 0.05). In addition, a similar degree of α-syn-ir aggregate-like profiles (Fig. 6G and H) and swollen dystrophic THir axons (Fig. 6I and J) in the ipsilateral striatum of Active vs. Inactive rats was observed. Collectively, these results suggest that Active STN DBS does not mitigate α-syn overexpression-mediated loss of THir terminal density in the striatum.

#### STN DBS increases both rpS6 and phosphorylated rpS6 immunoreactivity

**Figure 7:**
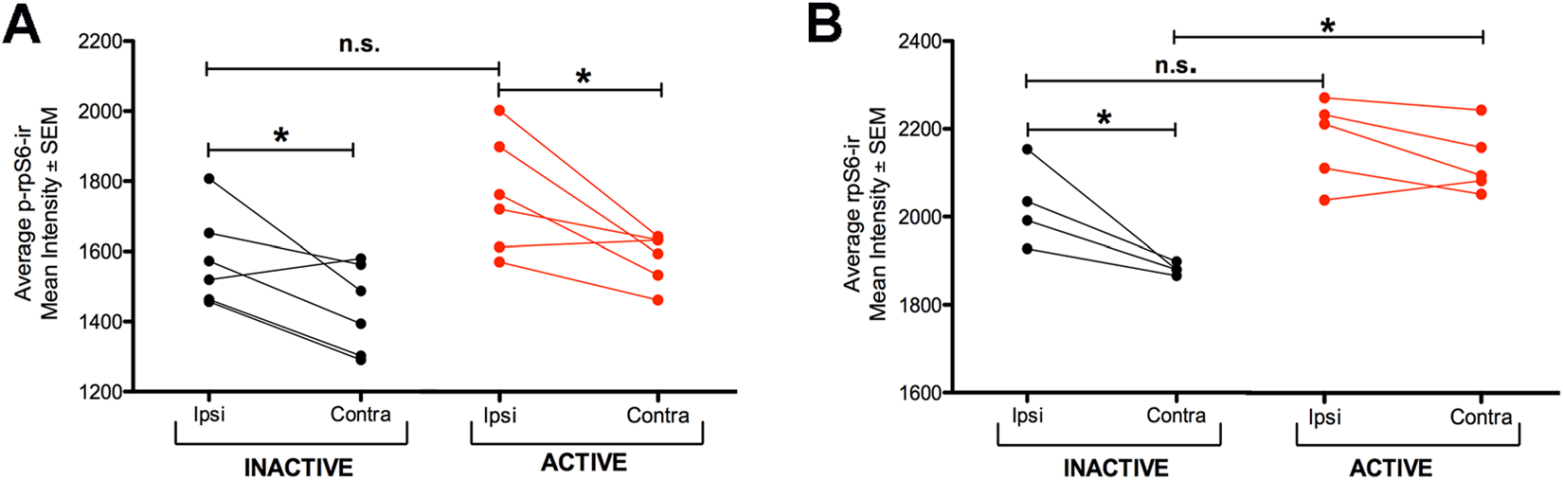
Effects of STN DBS on Phosphorylated and Total Levels of rpS6. Quantification of the Inactive and Active groups was assessed between hemispheres and treatments to determine the effect of stimulation on p-rpS6 (**A**) and rpS6 (**B**). (**A)** THir SN neurons expressing hu-α-syn exhibited significantly more p-rpS6 immunoreactivity (Inactive, n = 6: t_(5)_ = 2.805, *p* = 0.0378; Active, n = 6: t_(5)_ = 3.040, *p* = 0.0287). However, p-rpS6 appeared to increase slightly within α-syn expressing THir SN neurons in association with stimulation, whereas STN DBS overall had no significant impact on p-rpS6 immunoreactivity in either the ipsilateral or contralateral SNpc (F_(3,22)_ = 5.715, *p* > 0.05). (**B**) Total rpS6 was significantly increased in THir SN neurons expressing hu-α-syn in Inactive rats (n = 4; *p* = 0.0491) but not in Active Rats (n = 6; *p* > 0.05). Active STN DBS did not affect rpS6 immunoreactivity in hu-α-syn expressing neurons (*p* > 0.05) but did increase rpS6 immunoreactivity in the contralateral SNpc THir neurons (*p* = 0.004).

In the No Electrode rats in *Experiment 1* we observed that modest α-syn overexpression is associated with a significant increase in p-rpS6 but not rpS6. Individual α-syn/THir and contralateral non-α-syn/THir SNpc neurons in Inactive and Active rats were similarly analyzed for levels of rpS6 and p-rpS6 immunoreactivity. As previously observed, THir SN neurons expressing hu-α-syn exhibited significantly more p-rpS6 immunoreactivity (Inactive: t_(5)_ = 2.805, *p* = 0.0378; Active: t_(5)_ = 3.04, *p* = 0.0287, Fig. 7A). However, p-rpS6 appeared to increase slightly within α-syn expressing THir SN neurons in association with stimulation, whereas STN DBS overall had no significant impact on p-rpS6 immunoreactivity in either the ipsilateral or contralateral SNpc (F_(3,22)_ = 5.715, *p* > 0.05; Fig. 7A). rpS6 also was significantly increased in THir SN neurons expressing hu-α-syn in Inactive rats (t_(3)_ = 3.206, *p* = 0.0491) but not in Active Rats (t_(4)_ = 1.708, *p* > 0.05) with no significant effect of Active STN DBS on rpS6 immunoreactivity in hu-α-syn expressing neurons observed (F_(3,18)_ = 10.105, *p* > 0.05; Fig. 7B). After post hoc comparisons, the only significant stimulation-induced treatment effect observed was an increase in rpS6 immunoreactivity in the contralateral SNpc THir neurons (*p* = 0.004). These results indicate that STN DBS does not elevate p-rpS6 levels in THir SN neurons expressing hu-α-syn above the increase that is already produced by modest α-syn overexpression.

#### STN DBS ‘ON’ Exacerbates Forelimb Asymmetry

**Figure 8:**
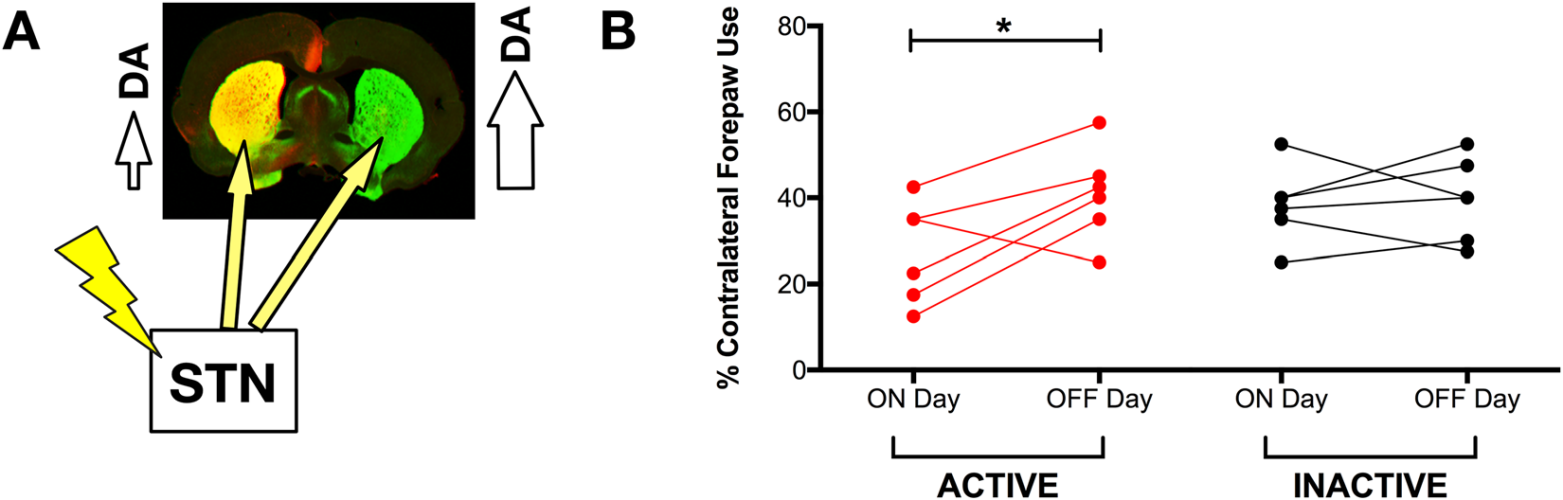
Acute STN Stimulation Exacerbates Forelimb Asymmetry. Unilateral overexpression of α-syn results in DA neurotransmission and handling dysfunction in the ipsilateral striatum and a functional impairment that was exaggerated by unilateral STN stimulation and an asymmetric release of striatal DA (**A**). (**B**) Forelimb use asymmetry was augmented by stimulation when compared to asymmetry in an off-stimulation cylinder task (n = 6; *p* = 0.047) but asymmetry was not exacerbated in the Inactive group (n = 6; *p* > 0.05).

In rats with near complete 6-OHDA-induced unilateral denervation of the striatum, acute STN DBS reverses deficits in contralateral forelimb akinesia^11,36,38–44^. In contrast, analysis of the striatum ipsilateral to rAAV2/5 α-syn injection in our model indicates that the overwhelmingly majority of dopaminergic innervation and dopamine (DA) tissue content is maintained following rAAV2/5 α-syn injection (Figs 2 H, 6F and Table 1). In non-lesioned rats, *unilateral* STN DBS results in *bilateral* striatal DA release^48^. Therefore, we examined the effects of STN DBS ‘ON’ under the present experimental parameters in which the α-syn overexpressing hemisphere possesses ≈75% striatal innervation and ≈90% SNpc neurons, compared to the contralateral hemisphere. Forelimb use in Inactive and Active rats was assessed in the cylinder task on Day 54 in the ‘ON’ state (Inactive rats remained with stimulation OFF and Active rats remained with stimulation ON) and then after 24 hours of washout in ‘OFF’ on Day 55 (all rats in OFF state, Fig. 1). As expected, Inactive rats showed identical forelimb use between Day 54 and Day 55 (Fig. 8B). Specifically, Inactive rats in OFF used their contralateral forelimb ≈40% of the time on both days. In contrast, STN DBS ON in Active rats increased contralateral forelimb akinesia (t_(5)_ = 2.630, *p* = 0.047; Fig. 8B). Specifically, when stimulation was OFF, rats used their contralateral forelimb ≈40% of the time, and this use decreased to ≈20% when stimulation was turned ON. These results suggest that Active unilateral STN DBS exacerbates the asymmetry in striatal DA release between the α-syn transduced and non-transduced striatum, driving DA release in both striatal hemispheres and suggesting DA release deficiencies in the α-syn expressing hemisphere, analogous to direct findings from an earlier study^49^.

## Discussion

In the present experiment, we sought to examine whether STN DBS provides neuroprotection against the toxicity that results from modest overexpression of human, wildtype α-syn in the nigrostriatal system. This nigrostriatal α-syn expression was by design, modest, in an attempt to more closely approximate levels of α-syn in sporadic PD^50,51^. Modest overexpression of α-syn more closely resembles the transcriptional signature observed in SNpc neurons in sporadic PD, whereas higher levels of overexpression produce downregulation of multiple trophic signaling models, a downregulation that is not observed in sporadic PD^34,35^. After eight weeks of unilateral overexpression of α-syn in the nigrostriatal system, we observed modest deficits in contralateral forelimb use (≈20% reduction), decreased density of dopaminergic terminals in the striatum (≈25% reduction) and loss of THir nigral neurons (≈10% reduction), either of which may represent loss of TH phenotype only. Striatal neuropathology also was associated with nigrostriatal α-syn overexpression, as indicated by axonal swellings and dystrophic neurites^52^. Based on our previous experience in which no degeneration or forelimb impairments resulted from nigral injections of a higher titer of rAAV2/5 encoding green fluorescent protein (GFP), we are confident that the impact of rAAV2/5 α-syn injections is α-syn specific^32,53–55^. We implanted stimulating electrodes following α-syn transduction and applied continuous STN DBS during ongoing α-syn-mediated insult that normally, without intervention, would result in measurable nigrostriatal and functional deficits. We observed that under these α-syn transduction parameters, arguably producing modest neurotoxicity relative to other studies^32,56–68^, one month of continuous STN DBS failed to provide any level of nigrostriatal or functional neuroprotection. In particular, no impact of STN DBS on α-syn-mediated striatal axonopathy was observed, and forelimb use deficits were not affected.

Based on this study, it is reasonable to speculate that under conditions of greater α-syn overexpression, a similar absence of neuroprotection would be observed. However, our recent data^34^ and present results suggest that modest overexpression of α-syn in the nigrostriatal system triggers quite a distinct cellular response in the SNpc compared to higher levels of α-syn overexpression. Specifically, previous work has demonstrated that an ≈8 fold increase in human wildtype α-syn in the SNpc of rats downregulates transcript levels of *Th, Bdnf*, nuclear receptor related 1 protein (*Nr4a2*) and rat *Snca* and also decreases the ratio of p-rpS6 to rpS6^35,66^. Using the present, more modest α-syn overexpression parameters (≈50% increase based on resulting pathology and protein measurements^32,33^), we do not observe changes in transcript levels of *Th, Bdnf, Nr4a2* and rat *Snca* and observe an increase in p-rpS6^34^. Therefore the toxicity that we observe in our modest α-syn overexpression paradigm is independent of downregulation of *Th, Bdnf, Nr4a2* and p-rpS6.

With differences in α-syn transgene load driving different SNpc responses the question becomes: What level of α-syn overexpression is expected to most accurately recapitulate levels of α-syn mRNA? Our recent work examining α-syn expression levels in sporadic PD reveals that α-syn gene expression is not increased in the early stage of PD or in association with disease progression and that the majority of SNpc neurons in PD contain normal transcript levels of *Th, Bdnf and Nr4a2* ^34^. We similarly found that modest overexpression of α-syn results in no impact on these transcripts in contrast to the results described in the rat rAAV model by Decressac *et al*.^35^ This raises the possibility that marked overexpression of α-syn may not be a relevant model for human sporadic PD. The results of a new study in which STN DBS is shown to protect SNpc THir neurons from viral vector-mediated A53T α-syn nigrostriatal overexpression^69^ must be interpreted in this light. Although A53T α-syn transgene load was not calculated, the resulting 25% SNpc THir neuron degeneration over a similar interval as our paradigm suggests a greater transgene load than our study. The effects A53T α-syn overexpression exerted on striatal THir innervation, previously reported to result in significant reductions^70^, were not examined in the Musacchio *et al*.^69^ study, and α-syn-induced deficits in striatal dopamine were not reversed by STN DBS. This study, like many others using neurotoxicants^10–14^, similarly failed to demonstrate STN DBS-mediated neuroprotection of striatal dopaminergic terminals. The ability of STN DBS to provide protection against α-syn axonopathy induced by more marked α-syn overexpression remains unknown.

Our present results suggest that the mechanism(s) by which STN DBS is neuroprotective against neurotoxicants (6-OHDA and MPTP)^10–14^ is/are ineffective in protecting against modest α-syn overexpression-mediated toxicity. Our previous work demonstrates that STN DBS increases BDNF levels in the nigrostriatal system and BDNF-trkB signaling in nigral THir neurons and that blockade of trkB receptors abolishes STN DBS-mediated neuroprotection^36,37^. Specifically, STN DBS is associated with phosphorylation of AKT and rpS6 in SNpc neurons^37^. In the present study, we used p-rpS6 levels as a molecular marker for trkB activation and observed that overexpression of α-syn alone (in the absence of STN DBS) increases rpS6 phosphorylation. We hypothesize that this increase in p-rpS6 may reflect a compensatory response of the neuron to the modest ongoing α-syn insult, similar to previous reports of a compensatory increase in TH and markers of dopaminergic transmission in early synucleinopathy preclinical models, early-stage PD and non-symptomatic Leucine-rich repeat kinase 2 (*LRRK2*) mutation carriers^71–73^. Moreover, STN DBS is unable to further increase rpS6 phosphorylation in SNpc neurons overexpressing α-syn. Therefore, it is possible that α-syn overexpression renders nigral dopamine neurons unresponsive to enhanced augmented trophic BDNF-trkB signaling. It is also possible that BDNF-trkB signaling may be ineffective in counteracting the neurotoxicity produced by α-syn overexpression.

In our study, following cessation of stimulation, rats that received four weeks of continuous STN DBS did not exhibit improvements in forelimb akinesia relative to rats that had received no stimulation. This indicates that continuous STN DBS did not provide neuroprotection against the functional deficits in contralateral forelimb use resulting from α-syn overexpression. Ideally the STN DBS parameters used to examine neuroprotective potential should be functionally relevant and reverse a lesion induced motor deficit; however, we were unable to capture an acute improvement in forelimb akinesia in this study when STN DBS was turned ON and OFF. However, the STN DBS stimulation parameters that we used were identical to those used in our previous studies (frequency of 130 Hz, a pulse width of 60 μs and an intensity of 30–50 μA), parameters in which we measured current spread and modified implantation coordinates to avoid lesioning the STN and that produce a significant improvement in forelimb use in the 6-OHDA model^10,36,74^. Further, we know that the onset of stimulation in these α-syn overexpressing rats resulted in an identical pattern of orofacial and contralateral forepaw dyskinesia until the stimulation intensity settings were lowered. Thus, based on all available data and our previously observed functional improvements using identical stimulation parameters, we can speculate that the stimulation parameters used in the present study were appropriate.

At first glance our forelimb asymmetry results appear paradoxical since acute STN DBS is well documented to reverse deficits in contralateral forelimb akinesia^11,36,38–44^. However, our acute stimulation ON and OFF results can be explained by bilateral effects on dopamine release. Specifically, during active STN DBS forelimb asymmetry was exacerbated compared to the level of asymmetry when STN DBS was turned off. In previous studies in which unilateral STN DBS ameliorates contralateral forelimb akinesia, unilateral nigrostriatal denervation induced by a neurotoxicant is near complete (i.e., greater than 90% striatal terminal loss)^11,36,38–44^. In the present unilateral α-syn overexpression model, whole tissue DA levels remain identical between hemispheres in the absence of stimulation; however, it is likely that release of striatal DA is decreased within the α-syn-overexpressing striatum, as has been previously reported^49^. Thus, applied in this context, unilateral STN DBS that stimulates bilateral DA release would exaggerate the asymmetry in DA release between the two striatal hemispheres, leading to an increased preference for ipsilateral paw use (Fig. 8A).

Our results essentially highlight the need to understand which model, if any, has the strongest predictive validity to examine the neuroprotective potential of STN DBS in PD. It is clear from results from multiple laboratories that STN DBS can protect from oxidative stress-initiated insults (e.g., 6-OHDA, MPTP)^10–14^, and work from our laboratory suggests that this effect is mediated by BDNF-trkB signaling^37^. However, the oxidative stress-based PD models have failed to predict efficacy in PD clinical trials, and in almost all instances these models lack α-syn pathology. Based on these limitations and our expanding knowledge of the central role of α-syn in both the genetic and idiopathic forms of PD, the PD research field has embraced viral vector-mediated α-syn overexpression as a preclinical model to more closely approximate pathogenic mechanisms in PD. Yet we are also beginning to appreciate that not all α-syn overexpression paradigms are equal and that transgene load may dictate mechanisms of toxicity. Another important consideration is a feature of the α-syn overexpression models that is not mirrored in sporadic PD and has received little attention: specifically, unlike the rare duplication/triplication mutations in *Snca* in which α-syn levels are elevated, α-syn mRNA is not increased in human sporadic PD^34,50,51^. It is possible that the induced, supraphysiological expression of α-syn, whether modest or marked, introduces a pathogenic mechanism(s) not relevant to sporadic PD. Indeed, the α-syn protein levels that have been achieved using the viral vector-mediated approach can be markedly higher than levels observed in cases of α-syn duplication and triplication mutations^75,76^. Our results demonstrate that STN DBS does not afford neuroprotection for the nigrostriatal system against modest α-syn overexpression-mediated toxicity. Whether STN DBS can provide neuroprotection from synucleinopathy in the context of normal levels of α-syn and human α-syn-related pathologic mechanisms remains to be determined. Ultimately, a clinical trial in which STN DBS is started early enough to modify the disease process will be the definitive test.

## Methods

### Animals

A total of 23, male, Sprague-Dawley rats (Harlan, ≈250 g) were used in this study. Rats were only included in the final analysis if they: (a) successfully completed the full stimulation interval, (b) displayed adequate electrode placement targeting the STN, as described previously^74^ and (c) had confirmation of viral vector transduction post mortem. Animals were allowed food and water *ad libitum* and were housed in reverse dark-light cycle conditions in an AAALAC approved facility. The Michigan State University Institutional Animal Care and Use Committee approved this study. All experiments were performed in accordance with relevant guidelines and regulations.

### Production of Recombinant Adeno-Associated Viral Vectors

The production of the α-syn-expressing, recombinant adeno-associated viral vector pseudotype 2/5 (rAAV2/5-α-syn) was conducted as described previously, and the exact same viral genome was used^32,77^; however, vector packaging was conducted in our laboratories at Michigan State University. Briefly, human cDNA was used to clone the α-syn coding sequence which was inserted into an AAV2 plasmid backbone under the control of the chicken beta actin/cytomegalovirus enhancer-promoter hybrid. Vector production was accomplished via co-transfecting HEK 293 T cells with the genome encoding plasmid together with a plasmid containing AAV2 rep and AAV5 cap genes and adenovirus helper functions. Cells were harvested 72 hours later and viral particles were purified using an iodixanol gradients followed by q-sepharose chromatography. Vector titers were determined using dot blot^78–80^. The viral vectors were stored at 4 °C and were never frozen. Surfaces in contact with virus were coated beforehand with Sigmacote (Sigma-Aldrich, St. Louis, MO). The rAAV2/5-α-syn titer used in this study was 1.8 × 10^12^ genome copies per ml.

### Intranigral Vector Injections

Intranigral vector injections were conducted as described previously^32^. Prior to surgery, anesthesia was induced with 5% isoflurane in O_2_, and rats were maintained under anesthesia with 2% isoflurane in O_2_. Rats received two unilateral, intranigral injections (AP −5.3 mm, ML + 2.0 mm, DV −7.2 mm and AP −6.0 mm, ML + 2.0 mm, DV −7.2 mm relative to dura mater) of rAAV2/5-α-syn (injection rate 0.5 μl/minute, 2.0 μl per site). These rAAV2/5 vector injection parameters have been demonstrated to result in transduction of >30% of SNpc THir neurons^53,54^.

### Electrode Implantation

Rats assigned to the Inactive and Active groups were implanted with electrodes eighteen days following vector surgery. This time point corresponds to the timing of near maximal α-syn protein expression^81^ but prior to significant deficits in THir terminal density or THir SNpc neuron loss^32^. Rats were anesthetized prior to surgery with Equithesin (0.3 ml**/**100 g body weight i.p.; chloral hydrate 42.5 mg/ml + sodium pentobarbital 9.72 mg/ml); they were subsequently, unilaterally implanted (ipsilateral to vector injections) with a bipolar, concentric microelectrode (inner electrode projection 1.0 mm, inner insulated electrode diameter 0.15 mm, outer electrode gauge 26, Plastics One, Roanoke, VA) targeted to the dorsal border of the STN (AP −3.4 mm, ML + 2.5 mm, relative to bregma and DV −7.7 mm, relative to the dura mater). Burr holes were drilled in the skull; the electrode was fixed in place using bone screws and dental acrylic. Electrodes were lowered to coordinates corresponding to the dorsal border of the STN in order to minimize damage to the nucleus.

### Behavioral Testing

Spontaneous forelimb use was assessed using the cylinder task as described previously^36,82,83^. Other behavioral measures were not employed due to their incompatibility with the external hardware required for continuous stimulation in awake animals. The cylinder task was employed at the following times: (a) prior to vector surgery, (b) two weeks following vector surgery and before electrode implantation, (c) 54 days following vector surgery, on stimulation and (d) 55 days following surgery, OFF stimulation. After testing OFF stimulation, the stimulators were re-started until sacrifice.

During the dark cycle, rats were videotaped and placed in a clear plexiglass cylinder until twenty, weight-bearing forelimb placements on the side of the cylinder occurred, or until a maximum trial time of five minutes had elapsed. To determine if forelimb preference was present, the number of contralateral, ipsilateral, and simultaneous paw placements was quantified. Data are reported as the percentage of contralateral (to vector and electrode) forelimb use: [(contralateral + 1/2 both)/(ipsilateral + contralateral + both)] × 100%. Rats with a unilateral, nigrostriatal lesion will show a bias toward using the ipsilateral limb.

### Continuous Stimulation Paradigm

When rats were assigned to receive stimulation (viz., the ‘Active’ group), stimulation was continuously delivered starting on Day 28 in a freely moving setup as previously described^10^. The start of stimulation was timed to occur prior to significant deficits in THir terminal density or THir SNpc neuron loss. Stimulation was generated by an Accupulser Signal Generator (World Precision Instruments, Sarasota, FL) via a battery-powered Constant Current Bipolar Stimulus Isolator (World Precision Instruments, Sarasota, FL). Stimulation parameters consisted of a frequency of 130 Hz, a pulse width of 60 μs and an intensity of 30–50 μA. At the onset of stimulation, intensity settings were increased until orofacial or contralateral forepaw dyskinesias were observed in order to confirm stimulation delivery, and immediately following a positive dyskinetic response, the intensity was set below the lower limit of dyskinesias, such that no rat was functionally impaired by stimulation. When rats were not being stimulated, they were still physically connected within their stimulator bowls to a commutator for the duration of the behavioral task.

### Euthanasia and Sectioning

At eight weeks (or 56 days) post vector surgery, stimulation was ceased, and rats were deeply anesthetized (60 mg/kg, pentobarbital, intraperitoneal) and perfused intracardially with heparinized normal saline at 37 °C followed by ice-cold paraformaldehyde (PFA) for 19 rats or ice-cold saline for 4 rats for HPLC and qPCR analyses of the striatum and SN, respectively. Care was taken to minimize the tissue damage resulting from removing the electrode from the skull. All 19 brains were post-fixed in 4% paraformaldehyde for twenty-four hours and transferred to 30% sucrose in 0.1 M phosphate buffer. PFA-perfused and postfixed brains were frozen on dry ice and sectioned at 40 μm thickness using a sliding microtome in six series.

### α-Synuclein Immunohistochemistry

One series (i.e., every sixth section) was stained with antisera for α-synuclein (α-syn) using the free-floating method, as described previously^32^. Tissue was blocked in normal goat serum and incubated overnight in primary antisera directed against wild-type human α- syn (mouse monoclonal anti-human α-syn, Invitrogen AHB0261, 1:2000 dilution for final concentration of 250 ng/ml) in 1.0% normal goat serum (Gibco, Catalog #16210–072). Cell membranes were permeabilized with the addition of Triton-X (0.5%, Sigma X-100) to the 0.1 M Tris buffer during incubations. Sections were then incubated in biotinylated secondary antisera against mouse IgG (Chemicon AP124B, 1:400 dilution for final concentration of 7.5 µg/ml) and followed by the Vector ABC detection kit employing horseradish peroxidase (Vector Laboratories, Burlingame, CA). α-Syn immunoreactive (α-syn-ir) neurons were visualized upon exposure to 0.5 mg/ml 3,3′-diaminobenzidine (DAB) and 0.03% H_2_O_2_ in tris-buffered saline (TBS). Sections were mounted on subbed slides, dried flat overnight under standard temperature and pressure conditions, dehydrated with ethanol and then xylenes and finally coverslipped with Cytoseal (Richard-Allan Scientific, Waltham, MA).

### Combined α-Synuclein and Tyrosine Hydroxylase Immunohistochemistry for Near- Infrared Imaging and Optical Density Analysis

One series (i.e., every sixth section) was stained with both antisera for α-syn and antisera for TH using the free-floating method, as previously described^32^. Tissue was blocked in Odyssey blocking buffer (LI-COR Bioscience, Lincoln, NE, 927–40000) with 0.5% Triton-X 100 (Sigma, X-100) at room temperature for one hour followed by overnight incubation in primary antisera directed against TH (rabbit anti-TH antibody, Millipore, Catalog #AB152, 1:1000 dilution) at 4 °C. Tissue was then washed for one hour (6 × 10 min) in 0.1 M tris-buffered saline (TBS) and incubated in secondary antisera (LiCOR donkey anti-rabbit 800 Catalog #926-32213, 1:500 dilution in blocking buffer) for one hour at room temperature; from this point forward, sections were covered or were kept in a dark room in order to minimize exposure to light. Sections subsequently were rinsed in 0.1 M TBS for forty minutes (4 × 10 min) and blocked again in blocking buffer with 0.5% Triton X-100 for one hour. Sections were incubated overnight in primary antisera directed against wild-type human α-syn (mouse anti-human a-syn antibody, Invitrogen Catalog #AHB0261, 1:2000 dilution for final concentration of 250 ng/ml) at 4 °C. Tissue was then washed for one hour (6 × 10 min) in 0.1 M TBS and incubated in secondary antisera (LI-COR donkey anti-mouse 680, Catalog #926-68022, 1:500 dilution for final concentration of 2 μg/ml) for one hour at room temperature. Lastly, sections were rinsed for forty minutes (4 × 10 min) in 0.1 M TBS. Sections were mounted on subbed slides, dried flat overnight under standard temperature and pressure conditions, dehydrated with ethanol and then xylenes and finally coverslipped with Cytoseal (Richard-Allan Scientific, Waltham, MA).

### Densitometry

A LI-COR Odyssey near-infrared scanner (LI-COR Biosciences) was used to scan every sixth section that was fluorescently labeled for both TH and α-syn using different wavelengths. In order to determine if any unilateral changes in TH or α-syn expression occurred within our groups, the integrated signal intensities were measured for both the ipsilateral and contralateral striatal hemispheres on slides normalized to a background measurement taken of the dorsolateral cortex on the contralateral side. The most rostral and caudal sections used were located AP + 2.28 mm and AP −0.24 mm, respectively^84^. The ipsilateral hemisphere was identified by verifying human α-syn expression. For each striatal section analyzed, a dorsolateral, ‘pie-shaped’ region of interest was defined as described previously^32^ due to involvement in forepaw motor function^85–87^; briefly, the striatum was bisected with a vertical line for the medial boundary, and a horizontal line extending from the base of the ipsilateral lateral ventricle served as the ventral boundary. The raw integrated intensity values (arbitrary units as defined by the software) from each sampled striatal section were averaged in order to mitigate any differences in the number of sampling sites per animal.

### Dissection of striatum for HPLC analysis and SN for qPCR

Frozen brains were equilibrated at a temperature of −20 °C prior to dissection. Brains were mounted on metal chucks using Tissue-Tek O.C.T. (VWR, Vatavia, IL), sectioned on a cryostat to the striatum and punched to a depth of 1 mm using a 1.5 mm diameter tissue punch. Frozen, dissected striatal punches were placed individually in vials and stored at −80 °C until analysis. Brains were then sectioned back to the rostral face of the SNpc and punched to a depth of 1 mm using a 1 mm × 1 mm tissue punch modified into an oval shape. The nigral tissue punch was immediately placed in TRIzol (Invitrogen, Grand Island, NY), homogenized with a disposable pestle and stored at −80 °C.

### High performance liquid chromatography (HPLC)

Homogenized samples were analyzed as described previously^10,88,89^. Briefly, samples were sonicated into an antioxidant solution (0.4 N perchlorate, 1.34 mM EDTA and 0.53 mM sodium metabisulfite), and protein concentration was determined on a small aliquot using a bicinchoninic acid (BCA) assay (Pierce). Clarified samples were separated on a 250 × 4.6 mm Microsorb MV C8 100-5 column (Varian, Walnut Creek, CA) and simultaneously examined for dopamine (DA), homovanillic acid (HVA) 3,4-dihydroxyphenylacetic acid (DOPAC), 5-hydroxytryptophan (5-HT), 5-hydroxyindoleacetic acid (5-HIAA) and norepinephrine (NE). Compounds were detected using a 12-channel coulometric array detector (CoulArray 5200, ESA, Chelmsford, MA) attached to a Waters 2695 Solvent Delivery System (Waters, Milford, MA) under the following conditions: flow rate of 1 ml/min; detection potentials of 50, 175, 350, 400 and 525 mV; and scrubbing potential of 650 mV. The mobile phase consisted of a 5% methanol solution in distilled water containing 100 mM citric acid, 75 mM Na_2_HPO_4_, 80 μM heptanesulfonate monohydrate and sodium salt at a pH of 4.25. Data are expressed as ng/mg protein.

### Tyrosine Hydroxylase Immunohistochemistry

One series (i.e., every sixth section) was stained with antisera for tyrosine hydroxylase (TH) using the free-floating method. Tissue was blocked in serum and incubated overnight in primary antisera directed against TH (Chemicon MAB318, mouse anti-TH, 1:4000). Cell membranes were permeabilized with the addition of Triton-X (0.3%) to the 0.1 M Tris buffer during incubations. Sections were then incubated in biotinylated secondary antisera against mouse IgG (Chemicon AP124B, 1:400) and followed by the Vector ABC detection kit employing horseradish peroxidase (Vector Laboratories, Burlingame, CA). TH immunoreactive (THir) neurons were visualized upon exposure to 0.5 mg/ml 3,3′-diaminobenzidine (DAB) and 0.03% H2O2 in Tris buffer. Sections were mounted on subbed slides, dried flat overnight under standard temperature and pressure conditions, dehydrated with ethanol and then xylenes and finally coverslipped with Cytoseal (Richard-Allan Scientific, Waltham, MA).

### Unbiased Stereology of THir Neurons in the SNpc

The number of THir neurons in the SNpc ipsilateral and contralateral to vector injections was quantified using unbiased stereology with the optical fractionator principle. Using a Nikon Eclipse 80i microscope, Retiga 4000R (QImaging, Surrey, BC, Canada) and Microbrightfield StereoInvestigator software (Microbrightfield Bioscience, Burlingame, VT), THir neuron quantification was completed by drawing a contour around the SNpc borders at 4X, and THir neurons were counted according to stereological principles at 60X (NA 1.4); estimates of total counts per structure were extrapolated by the software. The Schmitz-Hof Coefficients of Error were less than or equal to 0.10 for all analyses.

### Kluver-Barrera Histology

Every sixth section of the subthalamic nucleus (STN) was stained using Kluver-Barrera histochemistry^90^ and coverslipped with Cytoseal (Richard-Allan Scientific, Waltham, MA) to evaluate for appropriate targeting of the electrode to the STN. Only rats with correctly positioned electrodes were included in the study. Electrode location was considered to be appropriate if the tip of the electrode was observed within 250 μm of the border of the STN within any of the sections based on previous studies in which current spread was determined^74^. Although the 26 G outer electrode tract was readily visible, no appreciable loss of cells within the STN was observed, confirming our previously unbiased stereology results^10^.

### RNA isolation, conversion to cDNA and qPCR

RNA extraction was performed using the RNA Clean and Concentrator kit (Zymo Research, Irvine, CA) and eluted into 15 μl H_2_O. RNA from tissue was then converted into cDNA using SuperScript VILO Master Mix (Life Technologies, Grand Island, NY). The RNA was assumed to be converted 100% to cDNA. PCR reactions were run in 30 μl using target specific, Taqman hydrolysis probes for the gene of interest and were normalized to *Gapdh* (Ref 4351317, Applied Biosystems/Life Technologies, Carlsbad, CA). Normalized gene expression was determined by differences in the cycle thresholds (Ct) between genes of interest and *Gapdh* (ΔCt) on a ABI 7500 qPCR System (Applied Biosystems). The viral vector-injected SN (“ipsilateral”) was examined for transcript expression of the transgene human, wildtype *Snca* (Applied Biosystems assay ID Hs01103386_m1). Robust expression was required on the ipsilateral side for inclusion in this experiment; no transgene expression was detected on the contralateral side. The SN was also examined for the following transcripts (Applied Biosystems Assay ID# following): rat, wildtype *Snca* (Rn00569821_m1), *Bdnf* (Rn02531967_s1), *Th* (Rn00562500_m1) and *Trk2* (Rn01441749_m1).

### Triple-Label Immunofluorescence for Tyrosine Hydroxylase, Human α-Synuclein and Ribosomal Protein S6 or its Phosphorylated Form

One series (i.e., every sixth section) was triple labeled with antisera for TH, hu-α-syn and either ribosomal protein S6 (rpS6) or phosphorylated rpS6 (p-rpS6, Ser235/236) using the free-floating method. Tissue was washed and blocked in Odyssey blocking buffer (LI-COR Bioscience, Lincoln, NE, 927–40000) for one hour and incubated overnight in primary antisera directed against TH (Abcam ab76442, chicken anti-TH, 1:1000 for a final concentration of 200 ng/ml), hu-α-syn (Abcam #ab6162, mouse anti-hu-α-syn, 1:2000 for a final concentration of 500 ng/ml) and either rpS6 (Cell Signaling #2217, rabbit anti-rpS6 1:500 for final concentration of 30 ng/ml) or p-rpS6 (Cell Signaling #2211, rabbit anti-p-rpS6 1:400 for final concentration of 47.5 ng/ml). Cell membranes were permeabilized with the addition of Triton-X (0.5%) to the 0.1 M Tris buffer during incubations. Sections were then incubated in secondary antisera conjugated to fluorophores against chicken IgG (Alexa Fluor 488, Life Technologies #A11039, 1:500 for a final concentration of 4 μg/ml), rabbit IgG (Alexa Fluor 568, Life Technologies #A11011, 1:500 for a final concentration of 4 μg/ml) and mouse IgG (Goat anti-mouse 680, 1:500 for a final concentration of 2 μg/ml, LI-COR #926-68070) for one hour and rinsed. Sections were mounted on subbed slides and coverslipped with Vectashield Hardset Mounting Medium (Vector Laboratories H1400). All treatment groups were processed simultaneously.

Quantification of immunofluorescent intensity was conducted at a cell-level analysis. The first three SNpc sections in which the fibers of the medial terminal nucleus of the accessory optic tract are identified were photographed at 20X at a single focal plane using a Nikon Eclipse microscope, and images were stitched using Nikon Elements software. Identical exposure times and settings within each channel were used across all sections that are statistically compared. Using the same analysis software, ROIs were drawn around every THir SNpc neuron with identifiable cell boundaries and evidence of nuclear pallor and with the other channels not viewed by the investigator. ROI mean intensity data were exported for all channels. Each ROI/neuron across the three tissue sections was treated as a technical replicate and averaged. Collected images were composed as figure panels in OmniGraffle (The Omni Group, Seattle, WA), and minor adjustments were made using Photoshop (San Jose, CA) to signal distribution to use the entire dynamic range.

### Statistical Analyses

Statistical analyses were performed using IBM SPSS Statistics (IBM, Armonk, NY) or GraphPad Prism (La Jolla, CA). Statistical significance for all cases was set at *p* < 0.05. Levene’s test was used to verify the equal variances assumption, and a correction was used as necessary. To analyze the impact of α-syn overexpression on forelimb akinesia over time, a one-way RM-ANOVA (Fig. 2F) followed by least significant difference (LSD) post hoc analysis was used. To compare the impact of STN DBS (Active vs. Inactive) on α-syn overexpression-mediated forelimb deficits (Fig. 6A), a split-plot RM-ANOVA followed by least significant difference post hoc analysis was used. To compare the effects of unilateral α-syn overexpression between hemispheres on various outcome measures within the same rat (Table 1, Figs 2H,K, 4I–K, 6E and F, 7A and B) a paired samples t-test was conducted. A paired samples t-test was also used to analyze the impact of stimulation OFF vs ON (Fig. 8B) in the same rat. Within subject comparisons are indicated by a connecting line. To compare the effects of No Electrode vs. Active vs. Inactive stimulation between rats on α-syn immunoreactivity (Fig. 5C) and the effect of Active vs. Inactive stimulation on SNpc neurons (Fig. 6E) and TH immunoreactivity in the striatum (Fig. 6F), a one-way ANOVA with least significant difference post hoc analyses was used. To compare the effect of Active vs. Inactive stimulation on total and phosphorylated rpS6 (Fig. 7A and B), differences in immunofluorescence that are not visually verifiable, a more stringent Bonferroni post hoc analysis was used following one-way ANOVA. Data collected by qPCR were compared between hemispheres using the Relative Expression Software Tool 384 (REST-384 version 2) calculation software for the relative expression in real-time PCR using Pair-wise fixed reallocation randomization test (Fig. 3)^91^. Statistical outliers were assessed using the Absolute Deviation from the Median (ADAM) method using the ‘very conservative’ criterion^92^.

### Data Availability

The datasets generated during and/or analyzed during the current study are available from the corresponding author on reasonable request.

## References

[1] Volkmann J, Volkmann J (2004). Deep brain stimulation for the treatment of Parkinson’s disease. Journal of Clinical Neurophysiology.

[2] Krack P (2003). Five-year follow-up of bilateral stimulation of the subthalamic nucleus in advanced Parkinson’s disease. New Engl J Med.

[3] Rodriguez-Oroz MC (2004). Efficacy of deep brain stimulation of the subthalamic nucleus in Parkinson’s disease 4 years after surgery: double blind and open label evaluation. J Neurol Neurosur Ps.

[4] Rodriguez-Oroz MC (2005). Bilateral deep brain stimulation in Parkinson’s disease: a multicentre study with 4 years follow-up. Brain.

[5] Hilker R (2005). Disease progression continues in patients with advanced Parkinson’s disease and effective subthalamic nucleus stimulation. Journal of neurology, neurosurgery, and psychiatry.

[6] Tagliati M, Martin C, Alterman R (2010). Lack of motor symptoms progression in Parkinson’s disease patients with long-term bilateral subthalamic deep brain stimulation. The International journal of neuroscience.

[7] Cheng HC, Ulane CM, Burke RE (2010). Clinical progression in Parkinson disease and the neurobiology of axons. Annals of neurology.

[8] Kordower JH (2013). Disease duration and the integrity of the nigrostriatal system in Parkinson’s disease. Brain.

[9] Charles D (2014). *Subthalamic nucleus deep brain stim*ulation in early stage Parkinson’s disease. Parkinsonism & related disorders.

[10] Spieles-Engemann AL (2010). Stimulation of the rat subthalamic nucleus is neuroprotective following significant nigral dopamine neuron loss. Neurobiology of disease.

[11] Maesawa S (2004). Long-term stimulation of the subthalamic nucleus in hemiparkinsonian rats: neuroprotection of dopaminergic neurons. J Neurosurg.

[12] Temel Y (2006). Protection of nigral cell death by bilateral subthalamic nucleus stimulation. Brain research.

[13] Harnack D (2008). Placebo-controlled chronic high-frequency stimulation of the subthalamic nucleus preserves dopaminergic nigral neurons in a rat model of progressive Parkinsonism. Experimental neurology.

[14] Wallace BA (2007). Survival of midbrain dopaminergic cells after lesion or deep brain stimulation of the subthalamic nucleus in MPTP-treated monkeys. Brain.

[15] Thomas B, Muralikrishnan D, Mohanakumar KP (2000). *In vivo* hydroxyl radical generation in the striatum following systemic administration of 1-methyl-4-phenyl-1,2,3,6-tetrahydropyridine in mice. Brain research.

[16] Obata T, Yamanaka Y, Kinemuchi H, Oreland L (2001). Release of dopamine by perfusion with 1-methyl-4-phenylpyridinium ion (MPP(+)) into the striatum is associated with hydroxyl free radical generation. Brain research.

[17] Maharaj DS, Saravanan KS, Maharaj H, Mohanakumar KP, Daya S (2004). Acetaminophen and aspirin inhibit superoxide anion generation and lipid peroxidation, and protect against 1-methyl-4-phenyl pyridinium-induced dopaminergic neurotoxicity in rats. Neurochemistry international.

[18] Sanchez-Iglesias S, Rey P, Mendez-Alvarez E, Labandeira-Garcia JL, Soto-Otero R (2007). Time-course of brain oxidative damage caused by intrastriatal administration of 6-hydroxydopamine in a rat model of Parkinson’s disease. Neurochem Res.

[19] Smith MP, Cass WA (2007). Oxidative stress and dopamine depletion in an intrastriatal 6-hydroxydopamine model of Parkinson’s disease. Neuroscience.

[20] Themann C, Teismann P, Kuschinsky K, Ferger B (2001). Comparison of two independent aromatic hydroxylation assays in combination with intracerebral microdialysis to determine hydroxyl free radicals. Journal of neuroscience methods.

[21] Ferger B, Rose S, Jenner A, Halliwell B, Jenner P (2001). 6-hydroxydopamine increases hydroxyl free radical production and DNA damage in rat striatum. Neuroreport.

[22] Ferger B, Themann C, Rose S, Halliwell B, Jenner P (2001). 6-hydroxydopamine increases the hydroxylation and nitration of phenylalanine *in vivo*: implication of peroxynitrite formation. Journal of neurochemistry.

[23] Henze C (2005). Reactive oxidative and nitrogen species in the nigrostriatal system following striatal 6-hydroxydopamine lesion in rats. Brain research.

[24] NINDS NET-PD (2007). Investigators. A randomized clinical trial of coenzyme Q10 and GPI-1485 in early Parkinson disease. Neurology.

[25] de la Fuente-Fernandez R, Schulzer M, Mak E, Sossi V (2010). Trials of neuroprotective therapies for Parkinson’s disease: problems and limitations. Parkinsonism & related disorders.

[26] Marks WJ (2010). Gene delivery of AAV2-neurturin for Parkinson’s disease: a double-blind, randomised, controlled trial. Lancet neurology.

[27] Ibáñez P (2004). Causal relation between alpha-synuclein gene duplication and familial Parkinson’s disease. Lancet.

[28] Polymeropoulos MH (1997). Mutation in the alpha-synuclein gene identified in families with Parkinson’s disease. Science.

[29] Singleton AB (2003). alpha-Synuclein locus triplication causes Parkinson’s disease. Science.

[30] Spillantini MG (1997). Alpha-synuclein in Lewy bodies. Nature.

[31] Ulusoy A, Decressac M, Kirik D, Bjorklund A (2010). Viral vector-mediated overexpression of alpha-synuclein as a progressive model of Parkinson’s disease. Progress in brain research.

[32] Gombash SE (2013). Morphological and behavioral impact of AAV2/5-mediated overexpression of human wildtype alpha-synuclein in the rat nigrostriatal system. PloS one.

[33] Fischer DL (2016). Viral Vector-Based Modeling of Neurodegenerative Disorders: Parkinson’s Disease. Methods Mol Biol.

[34] Su, X. *et al*. Alpha-Synuclein mRNA Is Not Increased. *Molecular therapy: the journal of the American Society of Gene Therapy*, 10.1016/j.ymthe.2017.04.018 (2017). 10.1016/j.ymthe.2017.04.018 PMC5628770 28522034

[35] Decressac M (2012). alpha-Synuclein-induced down-regulation of Nurr1 disrupts GDNF signaling in nigral dopamine neurons. Science translational medicine.

[36] Spieles-Engemann AL (2011). Subthalamic Nucleus Stimulation Increases Brain Derived Neurotrophic Factor in the Nigrostriatal System and Primary MotorCortex. J Parkinsons Dis.

[37] Fischer, D. *et al*. Subthalamic Nucleus Deep Brain Stimulation Employs TrkB Signaling for Neuroprotection and Functional Restoration. *The Journal of neuroscience: the official journal of the Society for Neuroscience* (In Press). 10.1523/JNEUROSCI.2060-16.2017 PMC5508259 28607168

[38] Shi LH, Luo F, Woodward DJ, Chang JY (2006). Basal ganglia neural responses during behaviorally effective deep brain stimulation of the subthalamic nucleus in rats performing a treadmill locomotion test. Synapse.

[39] Meissner W (2002). Deep brain stimulation of subthalamic neurons increases striatal dopamine metabolism and induces contralateral circling in freely moving 6-hydroxydopamine-lesioned rats. Neuroscience letters.

[40] Chang JY, Shi LH, Luo F, Woodward DJ (2003). High frequency stimulation of the subthalamic nucleus improves treadmill locomotion in unilateral 6-hydroxydopamine lesioned rats. Brain research.

[41] Darbaky Y, Forni C, Amalric M, Baunez C (2003). High frequency stimulation of the subthalamic nucleus has beneficial antiparkinsonian effects on motor functions in rats, but less efficiency in a choice reaction time task. The European journal of neuroscience.

[42] Shi LH (2004). High-frequency stimulation of the subthalamic nucleus reverses limb-use asymmetry in rats with unilateral 6-hydroxydopamine lesions. Brain research.

[43] Fang X, Sugiyama K, Akamine S, Namba H (2006). Improvements in motor behavioral tests during deep brain stimulation of the subthalamic nucleus in rats with different degrees of unilateral parkinsonism. Brain research.

[44] Vlamings R (2007). High frequency stimulation of the subthalamic nucleus improves speed of locomotion but impairs forelimb movement in Parkinsonian rats. Neuroscience.

[45] Temel Y (2005). Acute and separate modulation of motor and cognitive performance in parkinsonian rats by bilateral stimulation of the subthalamic nucleus. Experimental neurology.

[46] Akita H, Honda Y, Ogata M, Noda K, Saji M (2010). Activation of the NMDA receptor involved in the alleviating after-effect of repeated stimulation of the subthalamic nucleus on motor deficits in hemiparkinsonian rats. Brain research.

[47] Li XH (2010). High-frequency stimulation of the subthalamic nucleus restores neural and behavioral functions during reaction time task in a rat model of Parkinson’s disease. Journal of neuroscience research.

[48] Bruet N (2001). High frequency stimulation of the subthalamic nucleus increases the extracellular contents of striatal dopamine in normal and partially dopaminergic denervated rats. Journal of neuropathology and experimental neurology.

[49] Lundblad, M., Decressac, M., Mattsson, B. & Bjorklund, A. Impaired neurotransmission caused by overexpression of alpha-synuclein in nigral dopamine neurons. *Proceedings of the National Academy of Sciences of the United States of America*10.1073/pnas.1200575109 (2012). 10.1073/pnas.1200575109 PMC3295273 22315428

[50] Kingsbury AE (2004). Alteration in alpha-synuclein mRNA expression in Parkinson’s disease. Movement disorders: official journal of the Movement Disorder Society.

[51] Neystat M (1999). Alpha-synuclein expression in substantia nigra and cortex in Parkinson’s disease. Movement disorders: official journal of the Movement Disorder Society.

[52] Chung CY, Koprich JB, Siddiqi H, Isacson O (2009). Dynamic changes in presynaptic and axonal transport proteins combined with striatal neuroinflammation precede dopaminergic neuronal loss in a rat model of AAV alpha-synucleinopathy. The Journal of neuroscience: the official journal of the Society for Neuroscience.

[53] Polinski NK (2015). Recombinant adenoassociated virus 2/5-mediated gene transfer is reduced in the aged rat midbrain. Neurobiology of aging.

[54] Polinski NK (2016). Impact of age and vector construct on striatal and nigral transgene expression. Mol Ther Methods Clin Dev.

[55] Gombash SE (2014). Neuroprotective potential of pleiotrophin overexpression in the striatonigral pathway compared with overexpression in both the striatonigral and nigrostriatal pathways. Gene Ther.

[56] Kirik, D. *et al*. Parkinson-like neurodegeneration induced by targeted overexpression of alpha-synuclein in the nigrostriatal system. *The Journal of neuroscience: the official journal of the Society for Neuroscience* **22**, 2780–2791, 20026246 (2002). 10.1523/JNEUROSCI.22-07-02780.2002 PMC6758323 11923443

[57] Gorbatyuk OS (2008). The phosphorylation state of Ser-129 in human alpha-synuclein determines neurodegeneration in a rat model of Parkinson disease. Proceedings of the National Academy of Sciences of the United States of America.

[58] Azeredo da Silveira S (2009). Phosphorylation does not prompt, nor prevent, the formation of alpha-synuclein toxic species in a rat model of Parkinson’s disease. Human molecular genetics.

[59] McFarland NR (2009). Alpha-synuclein S129 phosphorylation mutants do not alter nigrostriatal toxicity in a rat model of Parkinson disease. Journal of neuropathology and experimental neurology.

[60] Winner B (2011). *In vivo* demonstration that alpha-synuclein oligomers are toxic. Proceedings of the National Academy of Sciences of the United States of America.

[61] Yamada M, Iwatsubo T, Mizuno Y, Mochizuki H (2004). Overexpression of alpha-synuclein in rat substantia nigra results in loss of dopaminergic neurons, phosphorylation of alpha-synuclein and activation of caspase-9: resemblance to pathogenetic changes in Parkinson’s disease. Journal of neurochemistry.

[62] Yamada M, Mizuno Y, Mochizuki H (2005). Parkin gene therapy for alpha-synucleinopathy: a rat model of Parkinson’s disease. Human gene therapy.

[63] Ulusoy A, Febbraro F, Jensen PH, Kirik D, Romero-Ramos M (2010). Co-expression of C-terminal truncated alpha-synuclein enhances full-length alpha-synuclein-induced pathology. The European journal of neuroscience.

[64] Sanchez-Guajardo V, Febbraro F, Kirik D, Romero-Ramos M (2010). Microglia acquire distinct activation profiles depending on the degree of alpha-synuclein neuropathology in a rAAV based model of Parkinson’s disease. PloS one.

[65] Decressac M (2011). GDNF fails to exert neuroprotection in a rat alpha-synuclein model of Parkinson’s disease. Brain.

[66] Decressac M, Mattsson B, Lundblad M, Weikop P, Bjorklund A (2012). Progressive neurodegenerative and behavioural changes induced by AAV-mediated overexpression of alpha-synuclein in midbrain dopamine neurons. Neurobiology of disease.

[67] Decressac M, Mattsson B, Bjorklund A (2012). Comparison of the behavioural and histological characteristics of the 6-OHDA and alpha-synuclein rat models of Parkinson’s disease. Experimental neurology.

[68] Mulcahy P (2012). Development and characterisation of a novel rat model of Parkinson’s disease induced by sequential intranigral administration of AAV-alpha-synuclein and the pesticide, rotenone. Neuroscience.

[69] Musacchio, T. *et al*. STN-DBS is neuroprotective in the A53T alpha-synuclein Parkinson’s disease rat model. *Annals of neurology*, 10.1002/ana.24947 (2017). 10.1002/ana.24947 PMC5519923 28470693

[70] Koprich JB, Johnston TH, Reyes MG, Sun X, Brotchie JM (2010). Expression of human A53T alpha-synuclein in the rat substantia nigra using a novel AAV1/2 vector produces a rapidly evolving pathology with protein aggregation, dystrophic neurite architecture and nigrostriatal degeneration with potential to model the pathology of Parkinson’s disease. Molecular neurodegeneration.

[71] Volpicelli-Daley LA (2016). G2019S-LRRK2 Expression Augments alpha-Synuclein Sequestration into Inclusions in Neurons. The Journal of neuroscience: the official journal of the Society for Neuroscience.

[72] Paumier KL (2015). Intrastriatal injection of pre-formed mouse alpha-synuclein fibrils into rats triggers alpha-synuclein pathology and bilateral nigrostriatal degeneration. Neurobiology of disease.

[73] Adams JR (2005). PET in LRRK2 mutations: comparison to sporadic Parkinson’s disease and evidence for presymptomatic compensation. Brain.

[74] Spieles-Engemann AL, Collier TJ, Sortwell CE (2010). A functionally relevant and long-term model of deep brain stimulation of the rat subthalamic nucleus: advantages and considerations. The European journal of neuroscience.

[75] Farrer M (2004). Comparison of kindreds with parkinsonism and alpha-synuclein genomic multiplications. Annals of neurology.

[76] Volpicelli-Daley LA, Kirik D, Stoyka LE, Standaert DG, Harms AS (2016). How can rAAV-alpha-synuclein and the fibril alpha-synuclein models advance our understanding of Parkinson’s disease?. Journal of neurochemistry.

[77] Gombash SE (2012). Striatal Pleiotrophin Overexpression Provides Functional and Morphological Neuroprotection in the 6-Hydroxydopamine Model. Molecular therapy: the journal of the American Society of Gene Therapy.

[78] Benskey MJ, Sandoval IM, Manfredsson FP (2016). Continuous Collection of Adeno-Associated Virus from Producer Cell Medium Significantly Increases Total Viral Yield. Hum Gene Ther Methods.

[79] Benskey MJ, Manfredsson FP (2016). Intraparenchymal Stereotaxic Delivery of rAAV and Special Considerations in Vector Handling. Methods Mol Biol.

[80] Zolotukhin S (1999). Recombinant adeno-associated virus purification using novel methods improves infectious titer and yield. Gene Ther.

[81] Reimsnider S, Manfredsson FP, Muzyczka N, Mandel RJ (2007). Time course of transgene expression after intrastriatal pseudotyped rAAV2/1, rAAV2/2, rAAV2/5, and rAAV2/8 transduction in the rat. Molecular therapy: the journal of the American Society of Gene Therapy.

[82] Schallert, T. & Tillerson, J. L. in *Central Nervous System Diseases: Innovative Models of CNS Diseases from Molecule to Therapy Contemporary Neuroscience* (eds Dwaine F. Emerich, Reginald L. Dean III, & Paul R. Sanberg) Ch. 8, 131-151 (Humana Press, 2000).

[83] Schallert T (2006). Behavioral tests for preclinical intervention assessment. NeuroRx: the journal of the American Society for Experimental NeuroTherapeutics.

[84] Paxinos, G. & Watson, C. *The rat brain in stereotaxic coordinates*. 6th edn, (Elsevier, 2007).

[85] Brown LL (2002). Differential metabolic activity in the striosome and matrix compartments of the rat striatum during natural behaviors. The Journal of neuroscience: the official journal of the Society for Neuroscience.

[86] Kelley AE, Lang CG, Gauthier AM (1988). Induction of oral stereotypy following amphetamine microinjection into a discrete subregion of the striatum. Psychopharmacology (Berl).

[87] Pisa M (1988). Motor somatotopy in the striatum of rat: manipulation, biting and gait. Behav Brain Res.

[88] Perez SE (2005). Nigrostriatal dysfunction in familial Alzheimer’s disease-linked APPswe/PS1DeltaE9 transgenic mice. The Journal of neuroscience: the official journal of the Society for Neuroscience.

[89] Kanaan NM (2015). The longitudinal transcriptomic response of the substantia nigra to intrastriatal 6-hydroxydopamine reveals significant upregulation of regeneration-associated genes. PloS one.

[90] Kluver H, Barrera E (1953). A method for the combined staining of cells and fibers in the nervous system. Journal of neuropathology and experimental neurology.

[91] Pfaffl MW, Horgan GW, Dempfle L (2002). Relative expression software tool (REST) for group-wise comparison and statistical analysis of relative expression results in real-time PCR. Nucleic Acids Res.

[92] Leys C, Ley C, Klein O, Bernard P, Licata L (2013). Detecting outliers: Do not use standard deviation around the mean, use absolute deviation around the median. Journal of Experimental Social Psychology.

